# Expression of Annexin A4 in Cancer: A Tissue Microarray Study Involving More than 17,600 Cancers from 148 Tumor Entities

**DOI:** 10.3390/cancers18193077

**Published:** 2026-09-22

**Authors:** Cosima Völkel, Nayma Malas, Fiete Gehrisch, Anne Menz, Florian Lutz, Viktoria Chirico, Florian Viehweger, David Dum, Ria Schlichter, Andrea Hinsch, Christoph Fraune, Christian Bernreuther, Seyma Büyücek, Martina Kluth, Claudia Hube-Magg, Georgia Makrypidi-Fraune, Nina Schraps, Katharina Möller, Andreas M. Luebke, Patrick Lebok, Guido Sauter, Maximilian Lennartz, Till S. Clauditz, Andreas H. Marx, Ronald Simon, Eike Burandt, Natalia Gorbokon, Maria Christina Tsourlakis, Sarah Minner, Till Krech, Morton Freytag, Viktor Reiswich, Stefan Steurer

**Affiliations:** 1 Institute of Pathology, University Medical Center Hamburg-Eppendorf, Martinistr. 52, 20246 Hamburg, Germany; 2 Department of General, Visceral, Transplant and Thoracic Surgery, University Medical Center Hamburg-Eppendorf, 20246 Hamburg, Germany; 3 Institute of Pathology, Clinical Center Osnabrueck, 49076 Osnabrueck, Germany; 4 Department of Pathology, Academic Hospital Fuerth, 90766 Fuerth, Germany

**Keywords:** Annexin A4, ANXA4, cancer, immunohistochemistry, tissue microarray

## Abstract

**Background/Objectives:** Annexin A4 (ANXA4) is one of twelve members of the annexin superfamily that bind membrane phospholipids in a calcium-dependent manner, and which are involved in several processes related to the membrane. **Methods:** To learn more about the role of ANXA4 in cancer, the ANXA4 expression was analyzed by immunohistochemistry (IHC) on tissue microarrays (TMAs) containing 17,654 tumors and precursor lesions from 148 tumor types. **Results:** ANXA4 immunostaining was found in 10,858 (83.4%) of 13,017 analyzable tumors, and was considered weak in 10.1%, moderate in 16.6%, and strong in 61.8% of cases. Of 148 tumor categories, 144 showed ANXA4 staining in at least one case and 133 included at least one case with strong staining. Among tumor entities with at least 10 evaluable samples, strong ANXA4 staining in >50.0% of cases was seen in 56 of 120 entities, while absence of staining in >50.0% of cases was seen in 13. Clinically important tumor entities where ANXA4 staining was always positive (>99.5%) included gallbladder adenocarcinoma, cholangiocarcinoma, pancreatic/ampullary, various subtypes of salivary gland tumors, papillary renal cell carcinoma, urothelial carcinoma, squamous and basal cell carcinoma of the skin, and epithelioid mesothelioma. A reduced ANXA4 expression was linked to features of cancer aggressiveness in breast cancer, clear cell renal cell carcinoma, urothelial carcinoma, pancreatic ductal adenocarcinoma, hepatocellular carcinoma, colorectal adenocarcinoma, and follicular thyroid carcinoma. **Conclusions:** It is concluded that ANXA4 is abundantly expressed in human malignancies and that a reduced expression may be linked to unfavorable disease characteristics in many important tumor entities.

## Simple Summary

Annexin A4 (ANXA4), a member of the annexin superfamily is, involved in several processes related to the membrane. Using immunohistochemistry, ANXA4 expression was analyzed on tissue microarrays containing mor than 17,000 tumors and precursor lesions from 148 tumor types. Our results show that ANXA4 is abundantly expressed in human malignancies and reduced expression may be linked to unfavorable disease characteristics in many tumor entities.

## 1. Introduction

Annexin A4 (ANXA4) is one of twelve members of the annexin superfamily that bind membrane phospholipids in a calcium-dependent manner, providing a link between Ca-signaling and membrane functions [1,2]. Among the annexins, ANXA4 is one of the smallest proteins and is composed of only a core domain with short N-terminal regions [3]. ANXA4 is involved in several processes related to membrane dynamics such as membrane trafficking, vesicle aggregation, membrane organization, and ion channel regulation [3,4,5,6]. Consequently, ANXA4 is involved in numerous vital cellular processes including inflammation [7,8] and immunity [9,10], the regulation of cell growth [11] and apoptosis [12]. In normal tissues, ANXA4 is ubiquitously expressed and localized within the cytoplasm, cellular membranes, and nuclei across a wide range of cell types in most tissues [13,14]. ANXA4 dysregulation can contribute to abnormal cell migration, proliferation, and resistance to apoptosis [15].

The aberrant expression of ANXA4 has been implicated in cancer [13]. ANXA4 overexpression has been linked to tumor aggressiveness and treatment resistance in several cancer types [11,12,16,17,18,19,20,21,22,23,24,25,26,27]. Functional studies have demonstrated that ANXA4 upregulation can be sufficient to induce a malignant phenotype by promoting increased cellular proliferation, enhance migratory and invasive capabilities, and induce resistance to apoptosis, epithelial-mesenchymal transition, and altered cytoskeletal dynamics in cells of colorectal carcinoma [28], gastric cancer [11], ovarian cancer [12,26], and hepatocellular carcinoma (HCC) [29]. It has also been demonstrated that silencing ANXA4 results in a reduction in malignant cellular behavior in cells of lung cancer [22] and ovarian cancer [19,30]. Several authors have used immunohistochemistry (IHC) to assess the role of ANXA4 expression levels in various cancer types and found associations between both elevated and reduced ANXA4 protein levels and unfavorable disease parameters and a poor prognosis [12,16,17,18,19,20,21]. However, the published data are highly variable in different cancer types. For example, the range of reported ANXA4 positive cases ranged from 64.9% to 100% in colorectal adenocarcinoma [16,31], as well as from 25.0% to 66.7% in endometrioid [12,17], from 47.1% to 100.0% in mucinous [12,17], and from 8.3% to 35.2% in serous carcinoma of the ovary. Such a variability of data commonly occurs in IHC studies because of the use of different antibodies, staining protocols, and interpretation criteria [32].

To better understand the prevalence and role of ANXA4 in cancer, an extensive and highly standardized survey of ANXA4 protein expression in a broad range of different tumor types is needed. ANXA4 expression was thus evaluated in more than 13,000 tumor tissue samples and precursor lesions from 148 different tumor types and subtypes as well as 76 different non-neoplastic tissue types by IHC in a tissue microarray (TMA) format in this study.

## 2. Materials and Methods

### 2.1. Tissue Microarrays

Our normal tissue TMA was composed of 8 samples from 8 different donors for each of 76 different normal tissue types (608 samples on one slide). The cancer TMAs contained a total of 17,654 tumors and precursor lesions from 148 tumor types and subtypes. Detailed histopathological and molecular data were available for cancers of the breast (n = 1680), kidney (n = 1738), bladder (n = 535), pancreas (n = 598), stomach (n = 327), liver (n = 231), colorectum (n = 2351), endometrium (n = 182), ovary (n = 369), thyroid (n = 302), and testis (n = 565). The composition of both normal and cancer TMAs is described in detail in the Results Section. All samples were from the archives of the Institutes of Pathology, University Hospital of Hamburg, Germany; the Institute of Pathology, Clinical Center Osnabrueck, Germany; and Department of Pathology, Academic Hospital Fuerth, Germany. Tissues were fixed in 4% buffered formalin, and then embedded in paraffin. Representative tissue areas were selected by an experienced pathologist [33]. TMAs were composed of one tissue spot (diameter was 0.6 mm) per patient. The use of archived remnants of diagnostic tissues for manufacturing of TMAs and their analysis for research purposes as well as patient data analysis was according to local laws (HmbKHG, §12) and has been approved by the local ethics committee (Ethics commission Hamburg, WF-049/09). All work has been carried out in compliance with the Helsinki Declaration.

### 2.2. Immunohistochemistry

Freshly cut TMA sections were immunostained on one day and in one experiment. Slides were deparaffinized with xylol, rehydrated through a graded alcohol series, and exposed to heat-induced antigen retrieval for 5 min in an autoclave at 121 °C in pH 7.8 Tris-EDTA-Citrate (TEC) buffer. Endogenous peroxidase activity was blocked with Dako REAL Peroxidase-Blocking Solution (Agilent Technologies, Santa Clara, CA, USA; #S2023) for 10 min. Primary antibody specific for ANXA4 (recombinant mouse monoclonal antibody) (clone-ID ARX-504, MSVA GmbH, Hamburg, Germany; Cat# AO7830-504) was applied at 37 °C for 60 min at a dilution of 1:150. For the purpose of antibody validation, the normal tissue TMA was also analyzed by the ANXA4 mouse monoclonal antibody 1A10B5 (Protein tech, Rosemont, IL, USA; #60288-2Ig) at a dilution of 1:12,150 and an otherwise identical protocol. Bound antibody was then visualized using the Dako REAL EnVision Detection System Peroxidase/DAB+, Rabbit/Mouse kit (Agilent Technologies, Santa Clara, CA, USA; #K5007) according to the manufacturer’s directions. The sections were counterstained with hemalaun. For tumor tissues, the percentage of positive neoplastic cells was estimated, and the staining intensity was semi-quantitatively recorded (0, 1+, 2+, and 3+) by one experienced pathologist (SB), as previously described [34,35]. For statistical analyses, the staining results were categorized into four groups. Tumors without any staining were considered negative. Tumors with 1+ staining intensity in ≤70% of tumor cells or 2+ intensity in ≤30% of tumor cells were considered weakly positive. Tumors with 1+ staining intensity in >70% of tumor cells, 2+ intensity in 31–70%, or 3+ intensity in ≤30% of tumor cells were considered moderately positive. Tumors with 2+ intensity in >70% or 3+ intensity in >30% of tumor cells were considered strongly positive [36,37,38,39,40,41].

### 2.3. Statistics

All statistical calculations were performed with JMP18^®^ (18.2.1) software (SAS^®^, Cary, NC, USA). To explore univariate associations between ANXA immunostaining and tumor phenotype, contingency tables and the chi^2^-test were performed. Additional correction for multiple comparisons was performed using the Bonferroni method.

## 3. Results

### 3.1. Technical Issues

A total of 13,017 (73.7%) of 17,654 tumor samples were interpretable in our TMA analysis. Non-interpretable samples demonstrated a lack of unequivocal tumor cells or a complete lack of tissue.

### 3.2. ANXA4 in Normal Tissues

ANXA4 immunostaining occurred in most tissues and cell types. It was predominantly seen on cell membranes and in the cytoplasm, but it also occurred on nuclear membranes and in nuclei. The ANXA4 staining was most intense in epithelial cells of the gallbladder and of intrahepatic bile ducts, excretory and intercalated ducts of the pancreas, and on ciliae of epithelial cells of the fallopian tube and the epididymis. A strong ANXA4 staining was also seen in a subset of glandular cells in the stomach, epithelial cells of the small intestine (duodenum and ileum), Brunner glands, endometrium glands, the syncytiotrophoblast of the placenta, a fraction of pneumocytes, respiratory epithelium, and subsets of epithelial cells of the parathyroid. Nuclear ANXA4 staining was most conspicuous on gastric surface epithelium, and in chorion cells of the placenta. ANXA4 staining was weak or absent in adrenocortical cells, most subtypes of lymphocytes and of the cells of the bone marrow, ovary, testis (except Leydig cells), glandular cells of salivary glands, heart and skeletal muscle, as well as the brain. Representative images of ANXA4 immunostaining in different normal tissues are shown in Figure 1. All positive staining seen by ARX-504 were also confirmed by the mouse monoclonal antibody 1A10B5 and thus, considered specific (Supplementary Figure S1).

### 3.3. ANXA4 Immunostaining in Cancer

ANXA4 immunostaining predominated in the cytoplasm, but it was often also seen in membranes and rarely in nuclei and nuclear membranes of cancer cells. ANXA4 immunostaining was found in 10,858 (83.4%) of the 13,017 interpretable tumor samples, including 1310 (10.1%) with weak, 1510 (11.6%) with moderate, and 8038 (61.8%) with strong positivity (Table 1). Of 148 tumor entities, 144 showed ANXA4 staining in at least one case, while 133 tumor categories included at least one case with strong staining. Among tumor entities with at least 10 evaluable samples, strong ANXA4 staining in >50.0% of cases was observed in 56 (46.7%) of 120 entities, while an absence of staining in >50.0% of cases occurred in 13 (10.8%) entities. Among clinically important tumor entities, ANXA4 was always positive (>99.5%) in gallbladder adenocarcinoma, cholangiocarcinoma, pancreatic ductal and ampulla Vateri adenocarcinoma, various subtypes of salivary gland tumors, papillary renal cell carcinoma (pRCC), urothelial carcinoma of the kidney pelvis, squamous and basal cell carcinoma of the skin, and epithelioid mesothelioma. ANXA4 positivity among clinically important tumor entities was least frequent in lymphomas (positivity 7.1–46.2%), testicular seminoma (10.3%), osteosarcoma (30.8%), ovarian serous high-grade carcinoma (35.7%), mucinous carcinoma of the breast (42.9%), medullary thyroid carcinoma (44.0%), and invasive breast carcinoma of no special type (NST) (47.3%). Representative tumor images are shown in Figure 2. A graphical ranking of ANXA4-positive and strongly positive cancers is provided in Figure 3.

The relationship between ANXA4 immunostaining and histopathological parameters of cancer aggressiveness is summarized in Table 2. The results adjusted for multiple comparisons are presented in Supplementary Table S1. A reduced ANXA4 expression and/or loss of staining were associated with an advanced tumor stage (*p* < 0.0001), a high grade (*p* = 0.0018) and nodal metastasis (*p* = 0.0247) in breast cancer NST; a high ISUP (*p* = 0.0026), Fuhrman (*p* = 0.0006), and Thoenes grade (*p* = 0.0135), as well as a high UICC-stage (*p* = 0.0140) in clear cell renal cell carcinoma (ccRCC); an advanced tumor stage in urothelial carcinoma of the bladder (*p* = 0.0102);, an advanced tumor stage in pancreatic ductal adenocarcinoma (*p* = 0.0017); a high grade in HCC (*p* = 0.0247); and a high tumor stage in colorectal adenocarcinoma (*p* = 0.0098). ANXA4 expression was unrelated to the tumor phenotype in pRCC, gastric adenocarcinoma, endometrioid endometrial carcinoma, ovarian serous high-grade carcinoma, papillary thyroid carcinoma, and testicular seminoma.

## 4. Discussion

The successful analysis of 13,017 tumors across 148 different tumor categories revealed that ANXA4 can be abundantly expressed in most, if not all, tumor types. Approximately 90% of all entities showed at least one case exhibiting strong staining, while >45% demonstrated a strong staining pattern in more than 50% of all cancers. The widespread expression of ANXA4 across diverse tumor entities argues against a utility of ANXA4 IHC for the diagnostic discrimination of different tumor entities. Cancer types consistently exhibiting lower expression levels or a complete lack of ANXA4 staining were less common but included several clinically relevant tumors such as lymphomas, testicular seminoma, osteosarcoma, serous high-grade carcinoma of the ovary, medullary thyroid carcinoma, and invasive breast carcinoma NST. It is of note that the reported ANXA4 expressions for entities with few analyzable tumors should be interpreted with caution.

Although only a fraction of cancer entities included in our study had previously been analyzed by IHC, most reports describe similarly high frequencies of ANXA4-positive cases. For example, 100% positive tumors were previously described in 23 ccRCCs [42], 77.9–95.3% in 46–86 ovarian clear cell carcinomas [12,19,43], 44.6% in 123 invasive breast cancers NST [20], and 8.3–35.2% in 5–19 serous carcinomas of the ovary [12,17,19]. Other studies revealed an ANXA4 positivity of 64.9–100% in 6–268 colorectal adenocarcinomas [16,31], 47.1–100% in 4–17 mucinous carcinomas of the ovary [12,17,19], and 25.0–66.7% in 8–12 ovarian endometrioid carcinomas [12,17,19]. Due to the considerable variability of the published data and a lack of data in most tumor entities, a comprehensive ranking order of ANXA4 expression in human cancer could not have easily been compiled from the literature.

Clinical relevance for ANXA4 expression might be derived from its potential as a therapeutic target, which has been suggested by previous authors [21,44]. Mechanistically, ANXA4 has previously been implicated in various cancer-promoting pathways such as ERK1/2 [45], CDK1 [11], NF-κB [12,46], PI3K/AKT [11,15], and JAK-STAT [47], and has also been associated with the acquisition of chemoresistance [17,22,23,25,27,30]. Although advanced clinical data is lacking, preclinical studies have demonstrated that targeting ANXA4 can successfully reduce malignant cellular properties [21,44]. Our comprehensive data on the prevalence of ANXA4 expression in neoplasms demonstrates that patients with many different tumor entities could benefit from anti-ANXA4 therapies if these approaches should become safe and effective.

The large number of tumors in our cohort enabled us to investigate the associations between ANXA4 expression levels and the parameters of cancer aggressiveness in many different tumor types. Importantly, in the absence of functional, survival, or interventional data, our findings should be regarded as hypothesis-generating. That a reduced ANXA4 expression correlated with tumor progression in 6 out of 12 cases is consistent with several previous reports. For example, Xin et al. observed a gradual decrease in ANXA4 staining intensity with increasing tumor aggressiveness in a tissue microarray study involving 498 prostatic adenocarcinomas [48]. Sitek et al. reported a significantly reduced ANXA4 expression in pancreatic intraepithelial neoplasia and in ductal pancreatic adenocarcinoma as compared to normal pancreatic epithelium [49]. However, other authors have found associations between aggressive disease and a high ANXA4 expression in several tumor types [12,16,17,18,19,20]. It is of note that the annexin family of proteins are generally thought to be context-dependent regulators of tumor progression [50]. In line with this notion, the prognostic impact of ANXA4 might vary between tumor types. Moreover, it cannot be excluded that such reciprocal findings between entities could be due to methodological heterogeneity, including variations in IHC protocols, antibody sensitivity, and the thresholds defining positivity. Diverging roles are, for example, well-established for Annexin A1 (ANXA1) [51]. While in vivo studies demonstrate impaired tumor growth and metastasis in ANXA1 knockout mice, ANXA1 may also act as a tumor suppressor at early stages of tumorigenesis by protecting against DNA damage [51,52,53,54].

Given these methodological considerations, it is of note that the assay used in this study was extensively validated by comparing our IHC findings in normal tissues with data obtained by another independent anti-ANXA4 antibody and RNA data derived from three different publicly accessible databases “https://www.proteinatlas.org/ENSG00000196975-ANXA4/tissue (accessed on 10 January 2025)” [55,56,57,58]. This approach follows the recommendations of the International Working Group for Antibody Validation [59]. The identification of cross-reactive antibody binding to other proteins than the target protein represents the most significant challenge for antibody validation, which is hard to achieve in cell line experiments. As the tissues from 76 different normal tissue categories (8 donors each) will contain most cell types of adult humans, it is very likely that a near-complete spectrum of human proteins with a broad spectrum of posttranslational modifications was evaluated for potential cross-reactivities in our study. The validity of our assay was supported by the preferential detection of ANXA4 staining in the gallbladder, pancreas, small intestine (duodenum and ileum), and the stomach, which are the tissues with highest ANXA4 RNA expression and the largely absent staining in organs with very low or absent RNA staining such as testis, adrenal gland, lymph node, bone marrow, ovary, glandular cells of salivary glands, and heart and skeletal muscle, as well as the brain. That all cell types detected as ANXA4-positive by ARX-504 were also positive by the independent second antibody 1A10B5 (Supplementary Figure S1) represents further strong support for the assay specificity.

To increase the representativity of TMAs when compared to the analysis of whole sections and to account for intratumoral heterogeneity, it has previously been suggested that TMAs should contain multiple samples per tumor [60,61,62,63]. However, given that a 2.5 µm whole section with an area of 3 × 2 cm of a tumor measuring 5 cm in diameter represents only 0.0023% of the entire tumor mass, such an approach does not substantially increase representativity [63]. Importantly, while TMAs allow the analysis of a standardized tumor mass per patient and, therefore, provide a standardized representativity for IHC data, the quantity of the tumor tissue analyzed on whole sections varies considerably [63]. Notably, in a cohort of 553 breast cancers, Torhorst et al. found TMAs to be superior to whole sections for the detection of prognostic parameters [63,64].

It is concluded that ANXA4 is abundantly expressed in most human malignancies. While high rates of tumors with a low ANXA4 expression are confined to few tumor entities, a reduced expression may be associated with unfavorable disease characteristics in many important tumor entities. Should any ANXA4 targeted therapy become available in the future, many patients could potentially benefit from such approaches.

## Figures and Tables

**Figure 1:**
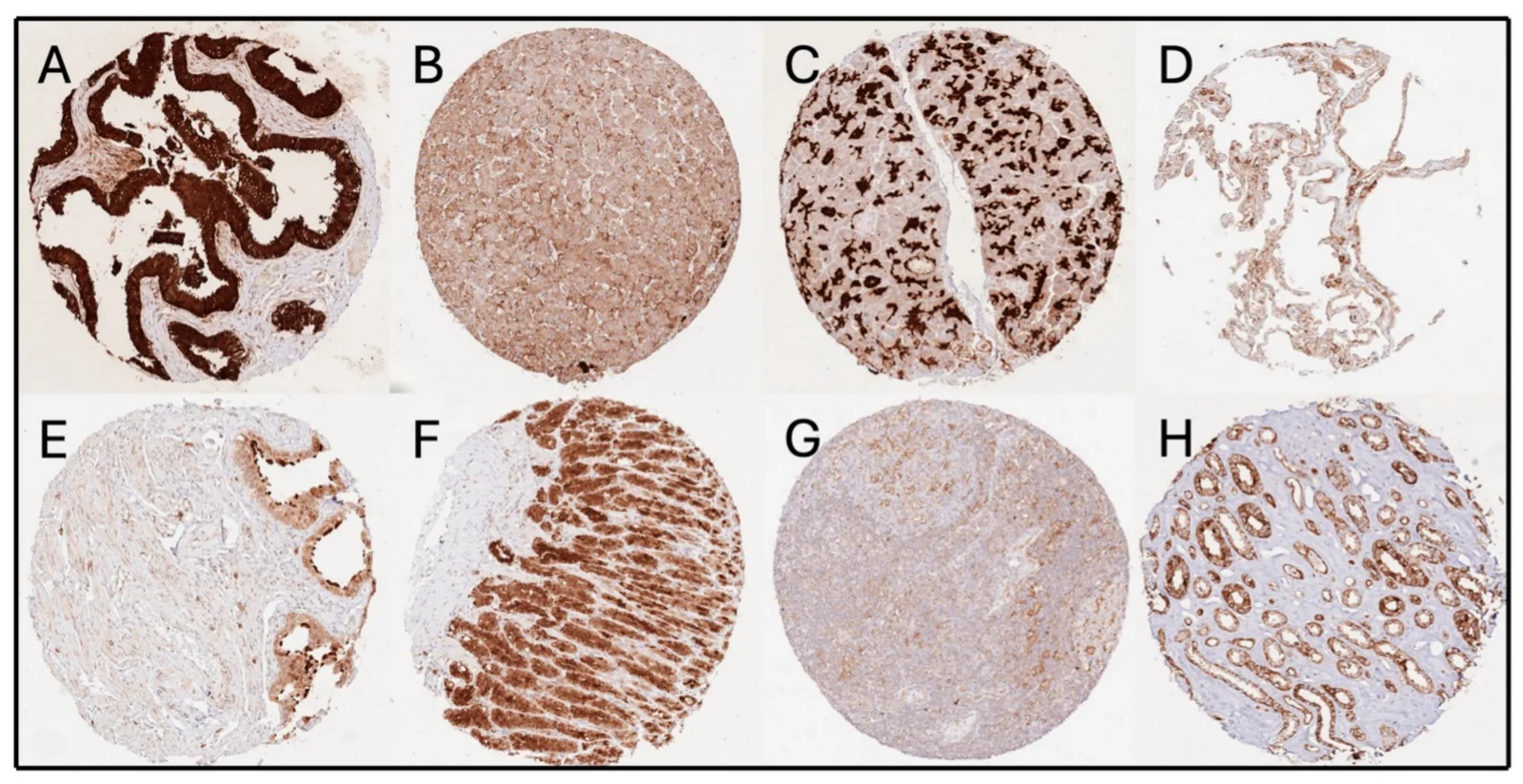
**ANXA4 immunostaining in normal tissue**. The panels show an intense ANXA4 staining in epithelial cells of the gallbladder (**A**), a moderate granular, cytoplasmic, and membranous staining of hepatocytes, as well as an intense staining of intrahepatic bile ducts in the liver (**B**), an intense cytoplasmic staining of intercalated ducts and excretory ducts of the pancreas (**C**), and a moderate staining of a subset of alveolar cells of the lung (**D**). ANXA4 staining is intense at ciliae, with moderate membranous and cytoplasmic staining of epithelial cells of the fallopian tube (**E**), moderate to strong in cells from the corpus glands of the stomach (**F**), moderate in monocytic sinus cells of a lymph node (**G**), and moderate to strong in collecting duct cells of the kidney (**H**).

**Figure 2:**
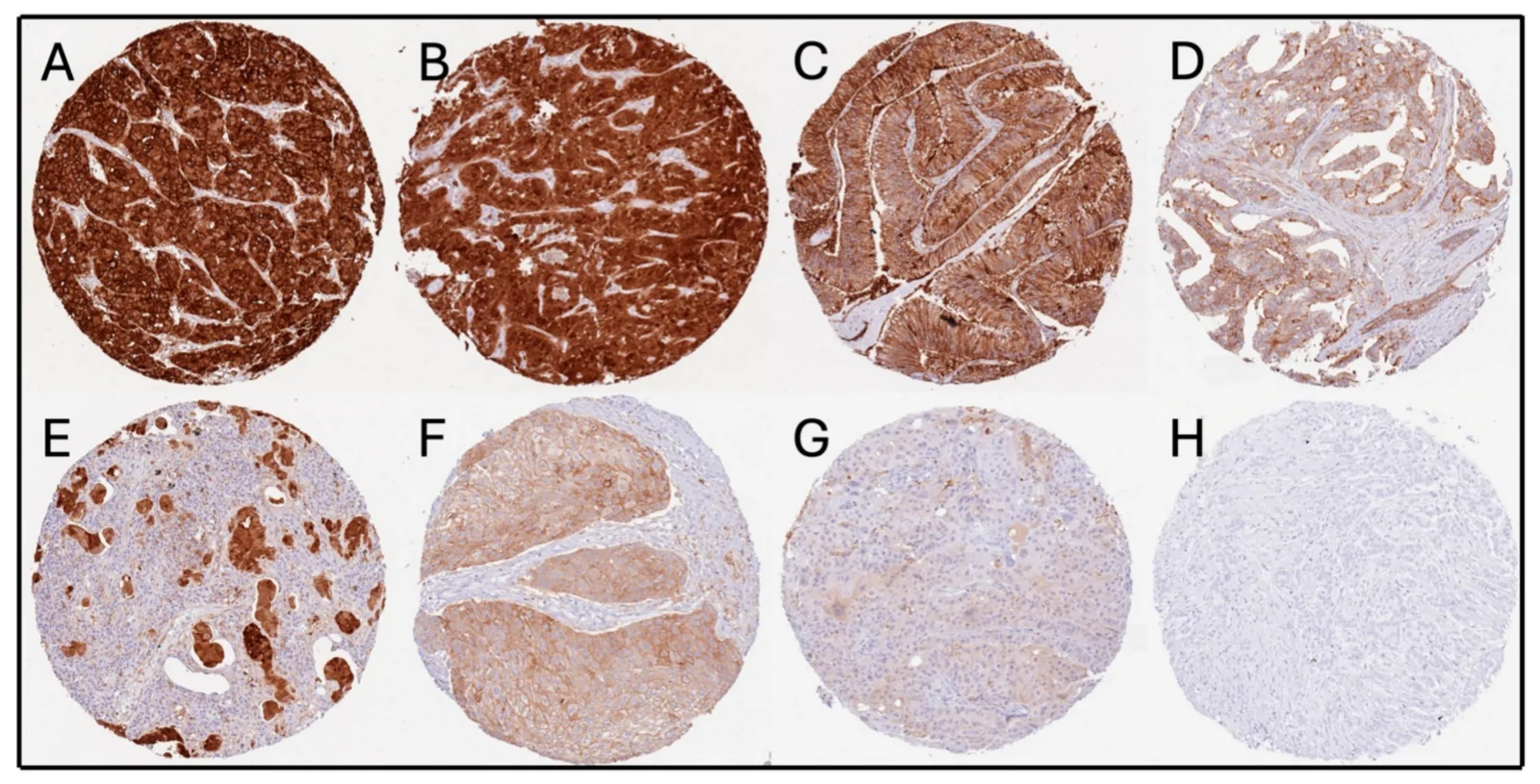
**ANXA4 immunostaining in cancer.** The panels show a strong cytoplasmic and membranous ANXA4 staining in a cholangiocarcinoma (**A**), a papillary renal cell carcinoma (**B**), and a colorectal adenocarcinoma (**C**), while the ANXA4 staining is moderate cytoplasmic and membranous in a prostatic adenocarcinoma ((**D**), Gleason 4 + 4), heterogeneous, weak to strong, and predominantly cytoplasmic in a pulmonary adenocarcinoma (**E**), weak to moderate, cytoplasmic, and membranous in a pharyngeal squamous cell carcinoma (**F**), and a serous high-grade carcinoma of the ovary (**G**). ANXA4 staining is absent in a breast carcinoma of no special type (**H**).

**Figure 3:**
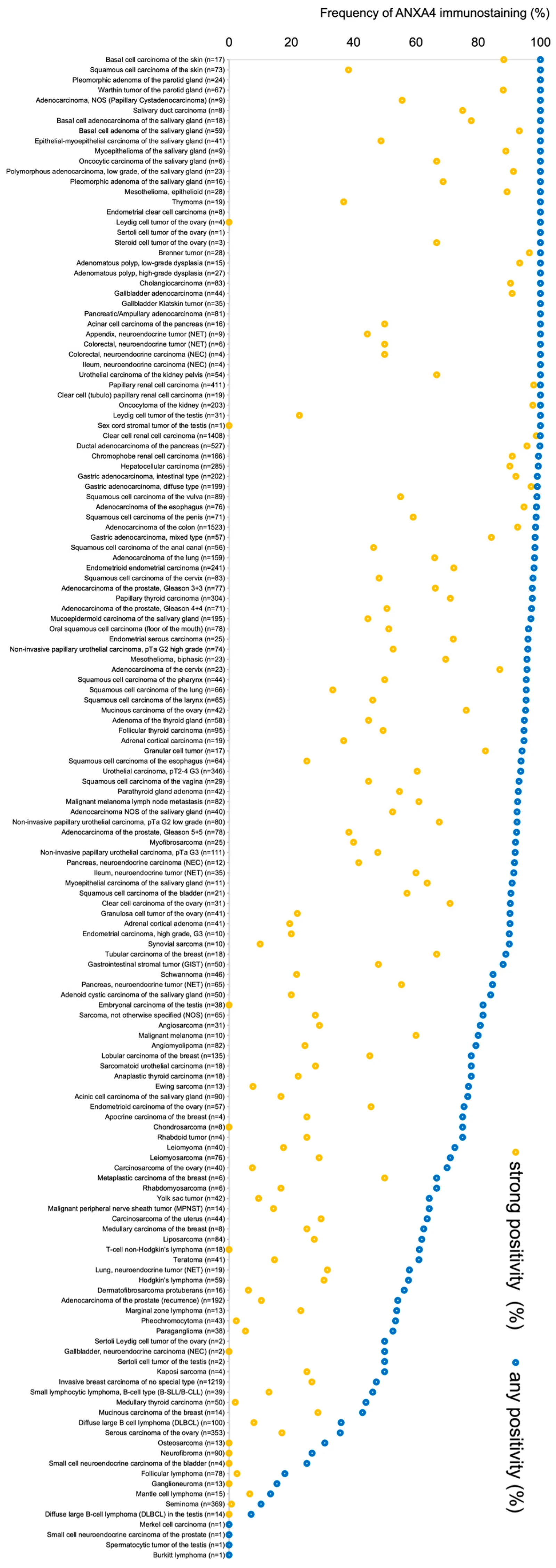
**Ranking order of ANX4 immunostaining in caner.** Ranking order of tumor entities according to their ANXA4 positivity rate. A blue dot represents the overall rate of positivity. A yellow dot represents the fraction of tumors with strong staining.

**Table 1:** ANXA4 immunostaining in cancer.

				Annexin A4 Immunostaining			
Tumor Category	Tumor Entity	on TMA (n)	Analyzable (n)	Negative (%)	Weak (%)	Moderate (%)	Strong (%)
**Tumors of the skin**	Basal cell carcinoma of the skin	89	17	0.0	5.9	5.9	88.2
	Squamous cell carcinoma of the skin	145	73	0.0	20.5	41.1	38.4
	Malignant melanoma	65	10	20.0	10.0	10.0	60.0
	Malignant melanoma lymph node metastasis	86	82	7.3	17.1	14.6	61.0
	Merkel cell carcinoma	2	1	100.0	0.0	0.0	0.0
**Tumors of the head and neck**	Squamous cell carcinoma of the larynx	109	65	4.6	16.9	32.3	46.2
	Squamous cell carcinoma of the pharynx	60	44	4.5	27.3	18.2	50.0
	Oral squamous cell carcinoma (floor of the mouth)	130	78	3.8	19.2	25.6	51.3
	Pleomorphic adenoma of the parotid gland	50	24	0.0	0.0	0.0	100.0
	Warthin tumor of the parotid gland	104	67	0.0	4.5	7.5	88.1
	Adenocarcinoma, NOS (Papillary Cystadenocarcinoma)	14	9	0.0	33.3	11.1	55.6
	Salivary duct carcinoma	15	8	0.0	12.5	12.5	75.0
	Acinic cell carcinoma of the salivary gland	181	90	23.3	46.7	13.3	16.7
	Adenocarcinoma NOS of the salivary gland	109	40	7.5	22.5	17.5	52.5
	Adenoid cystic carcinoma of the salivary gland	180	50	16.0	54.0	10.0	20.0
	Basal cell adenocarcinoma of the salivary gland	25	18	0.0	16.7	5.6	77.8
	Basal cell adenoma of the salivary gland	101	59	0.0	5.1	1.7	93.2
	Epithelial-myoepithelial carcinoma of the salivary gland	53	41	0.0	22.0	29.3	48.8
	Mucoepidermoid carcinoma of the salivary gland	343	195	3.1	29.2	23.1	44.6
	Myoepithelial carcinoma of the salivary gland	21	11	9.1	9.1	18.2	63.6
	Myoepithelioma of the salivary gland	11	9	0.0	11.1	0.0	88.9
	Oncocytic carcinoma of the salivary gland	12	6	0.0	0.0	33.3	66.7
	Polymorphous adenocarcinoma, low grade, of the salivary gland	41	23	0.0	8.7	0.0	91.3
	Pleomorphic adenoma of the salivary gland	53	16	0.0	12.5	18.8	68.8
**Tumors of the lung, pleura and thymus**	Adenocarcinoma of the lung	196	159	1.9	14.5	17.6	66.0
	Squamous cell carcinoma of the lung	80	66	4.5	27.3	34.8	33.3
	Mesothelioma, epithelioid	32	28	0.0	3.6	7.1	89.3
	Mesothelioma, biphasic	25	23	4.3	8.7	17.4	69.6
	Thymoma	29	19	0.0	5.3	57.9	36.8
	Lung, neuroendocrine tumor (NET)	29	19	42.1	21.1	5.3	31.6
**Tumors of the female genital tract**	Squamous cell carcinoma of the vagina	30	29	6.9	6.9	41.4	44.8
	Squamous cell carcinoma of the vulva	107	89	1.1	12.4	31.5	55.1
	Squamous cell carcinoma of the cervix	88	83	2.4	14.5	34.9	48.2
	Adenocarcinoma of the cervix	23	23	4.3	4.3	4.3	87.0
	Endometrioid endometrial carcinoma	288	241	2.1	12.4	13.3	72.2
	Endometrial serous carcinoma	36	25	4.0	8.0	16.0	72.0
	Carcinosarcoma of the uterus	57	44	36.4	15.9	18.2	29.5
	Endometrial carcinoma, high grade, G3	13	10	10.0	40.0	30.0	20.0
	Endometrial clear cell carcinoma	9	8	0.0	0.0	0.0	100.0
	Endometrioid carcinoma of the ovary	93	57	24.6	22.8	7.0	45.6
	Serous carcinoma of the ovary	530	353	64.3	11.0	7.6	17.0
	Mucinous carcinoma of the ovary	75	42	4.8	11.9	7.1	76.2
	Clear cell carcinoma of the ovary	51	31	9.7	12.9	6.5	71.0
	Carcinosarcoma of the ovary	47	40	30.0	37.5	25.0	7.5
	Granulosa cell tumor of the ovary	44	41	9.8	36.6	31.7	22.0
	Leydig cell tumor of the ovary	4	4	0.0	50.0	50.0	0.0
	Sertoli cell tumor of the ovary	1	1	0.0	0.0	0.0	100.0
	Sertoli Leydig cell tumor of the ovary	3	2	50.0	0.0	0.0	50.0
	Steroid cell tumor of the ovary	3	3	0.0	0.0	33.3	66.7
	Brenner tumor	32	28	0.0	0.0	3.6	96.4
**Tumors of the breast**	Invasive breast carcinoma of no special type	1396	1219	52.7	6.9	13.9	26.6
	Lobular carcinoma of the breast	159	135	22.2	7.4	25.2	45.2
	Medullary carcinoma of the breast	8	8	37.5	0.0	37.5	25.0
	Tubular carcinoma of the breast	20	18	11.1	11.1	11.1	66.7
	Mucinous carcinoma of the breast	18	14	57.1	7.1	7.1	28.6
	Apocrine carcinoma of the breast	4	4	25.0	50.0	0.0	25.0
	Metaplastic carcinoma of the breast	6	6	33.3	16.7	0.0	50.0
**Tumors of the digestive system**	Adenomatous polyp, low-grade dysplasia	50	15	0.0	0.0	6.7	93.3
	Adenomatous polyp, high-grade dysplasia	50	27	0.0	0.0	0.0	100.0
	Adenocarcinoma of the colon	2482	1523	1.6	2.5	3.3	92.6
	Gastric adenocarcinoma, diffuse type	215	199	1.0	0.5	1.5	97.0
	Gastric adenocarcinoma, intestinal type	215	202	1.0	2.5	4.5	92.1
	Gastric adenocarcinoma, mixed type	62	57	1.8	1.8	12.3	84.2
	Adenocarcinoma of the esophagus	83	76	1.3	2.6	1.3	94.7
	Squamous cell carcinoma of the esophagus	76	64	6.3	29.7	39.1	25.0
	Squamous cell carcinoma of the anal canal	91	56	1.8	19.6	32.1	46.4
	Cholangiocarcinoma	122	83	0.0	2.4	7.2	90.4
	Gallbladder adenocarcinoma	51	44	0.0	9.1	0.0	90.9
	Gallbladder Klatskin tumor	42	35	0.0	0.0	0.0	100.0
	Hepatocellular carcinoma	312	285	0.7	3.2	6.0	90.2
	Ductal adenocarcinoma of the pancreas	659	527	0.2	1.1	3.0	95.6
	Pancreatic/Ampullary adenocarcinoma	98	81	0.0	0.0	0.0	100.0
	Acinar cell carcinoma of the pancreas	18	16	0.0	12.5	37.5	50.0
	Gastrointestinal stromal tumor (GIST)	62	50	12.0	6.0	34.0	48.0
	Appendix, neuroendocrine tumor (NET)	25	9	0.0	22.2	33.3	44.4
	Colorectal, neuroendocrine tumor (NET)	12	6	0.0	33.3	16.7	50.0
	Ileum, neuroendocrine tumor (NET)	53	35	8.6	11.4	20.0	60.0
	Pancreas, neuroendocrine tumor (NET)	101	65	15.4	21.5	7.7	55.4
	Colorectal, neuroendocrine carcinoma (NEC)	14	4	0.0	50.0	0.0	50.0
	Ileum, neuroendocrine carcinoma (NEC)	8	4	0.0	0.0	0.0	100.0
	Gallbladder, neuroendocrine carcinoma (NEC)	4	2	50.0	50.0	0.0	0.0
	Pancreas, neuroendocrine carcinoma (NEC)	14	12	8.3	25.0	25.0	41.7
**Tumors of the urinary system**	Non-invasive papillary urothelial carcinoma, pTa G2 low grade	87	80	7.5	11.3	13.8	67.5
	Non-invasive papillary urothelial carcinoma, pTa G2 high grade	80	74	4.1	20.3	23.0	52.7
	Non-invasive papillary urothelial carcinoma, pTa G3	126	111	8.1	20.7	23.4	47.7
	Urothelial carcinoma, pT2-4 G3	416	346	6.4	10.4	22.8	60.4
	Squamous cell carcinoma of the bladder	22	21	9.5	4.8	28.6	57.1
	Small cell neuroendocrine carcinoma of the bladder	4	4	75.0	25.0	0.0	0.0
	Sarcomatoid urothelial carcinoma	23	18	22.2	27.8	22.2	27.8
	Urothelial carcinoma of the kidney pelvis	62	54	0.0	11.1	22.2	66.7
	Clear cell renal cell carcinoma	1730	1408	0.1	0.4	0.9	98.7
	Papillary renal cell carcinoma	513	411	0.0	0.7	1.5	97.8
	Clear cell (tubulo) papillary renal cell carcinoma	26	19	0.0	0.0	0.0	100.0
	Chromophobe renal cell carcinoma	207	166	0.6	1.8	6.6	91.0
	Oncocytoma of the kidney	247	203	0.0	0.0	2.5	97.5
**Tumors of the male genital organs**	Adenocarcinoma of the prostate, Gleason 3 + 3	83	77	2.6	7.8	23.4	66.2
	Adenocarcinoma of the prostate, Gleason 4 + 4	80	71	2.8	18.3	28.2	50.7
	Adenocarcinoma of the prostate, Gleason 5 + 5	85	78	7.7	25.6	28.2	38.5
	Adenocarcinoma of the prostate (recurrence)	258	192	45.8	29.2	14.6	10.4
	Small cell neuroendocrine carcinoma of the prostate	2	1	100.0	0.0	0.0	0.0
	Seminoma	680	369	89.7	7.9	1.6	0.8
	Embryonal carcinoma of the testis	43	38	18.4	52.6	28.9	0.0
	Leydig cell tumor of the testis	31	31	0.0	25.8	51.6	22.6
	Sertoli cell tumor of the testis	2	2	50.0	0.0	0.0	50.0
	Sex cord stromal tumor of the testis	1	1	0.0	100.0	0.0	0.0
	Spermatocytic tumor of the testis	1	1	100.0	0.0	0.0	0.0
	Yolk sac tumor	51	42	35.7	21.4	33.3	9.5
	Teratoma	52	41	39.0	29.3	17.1	14.6
	Squamous cell carcinoma of the penis	92	71	1.4	15.5	23.9	59.2
**Tumors of endocrine organs**	Adenoma of the thyroid gland	113	58	5.2	15.5	34.5	44.8
	Papillary thyroid carcinoma	391	304	2.6	6.9	19.4	71.1
	Follicular thyroid carcinoma	154	95	5.3	17.9	27.4	49.5
	Medullary thyroid carcinoma	111	50	56.0	26.0	16.0	2.0
	Parathyroid gland adenoma	43	42	7.1	2.4	35.7	54.8
	Anaplastic thyroid carcinoma	45	18	22.2	38.9	16.7	22.2
	Adrenal cortical adenoma	42	41	9.8	51.2	19.5	19.5
	Adrenal cortical carcinoma	27	19	5.3	26.3	31.6	36.8
	Pheochromocytoma	51	43	46.5	27.9	23.3	2.3
**Tumors of hematopoetic and lymphoid tissues**	Hodgkin’s lymphoma	103	59	42.4	1.7	25.4	30.5
	Small lymphocytic lymphoma, B-cell type (B-SLL/B-CLL)	50	39	53.8	25.6	7.7	12.8
	Diffuse large B cell lymphoma (DLBCL)	113	100	64.0	9.0	19.0	8.0
	Follicular lymphoma	88	78	82.1	11.5	3.8	2.6
	T-cell non-Hodgkin’s lymphoma	25	18	38.9	44.4	16.7	0.0
	Mantle cell lymphoma	18	15	86.7	6.7	0.0	6.7
	Marginal zone lymphoma	16	13	46.2	15.4	15.4	23.1
	Diffuse large B-cell lymphoma (DLBCL) in the testis	16	14	92.9	0.0	7.1	0.0
	Burkitt lymphoma	5	1	100.0	0.0	0.0	0.0
**Tumors of soft tissue and bone**	Granular cell tumor	19	17	5.9	0.0	11.8	82.4
	Leiomyoma	50	40	27.5	25.0	30.0	17.5
	Leiomyosarcoma	92	76	28.9	21.1	21.1	28.9
	Liposarcoma	92	84	38.1	21.4	13.1	27.4
	Malignant peripheral nerve sheath tumor (MPNST)	14	14	35.7	50.0	0.0	14.3
	Myofibrosarcoma	25	25	8.0	32.0	20.0	40.0
	Angiosarcoma	34	31	19.4	16.1	35.5	29.0
	Angiomyolipoma	85	82	20.7	31.7	23.2	24.4
	Dermatofibrosarcoma protuberans	20	16	43.8	43.8	6.3	6.3
	Ganglioneuroma	13	13	84.6	15.4	0.0	0.0
	Kaposi sarcoma	6	4	50.0	25.0	0.0	25.0
	Neurofibroma	100	90	73.3	25.6	1.1	0.0
	Sarcoma, not otherwise specified (NOS)	68	65	18.5	33.8	20.0	27.7
	Paraganglioma	40	38	47.4	28.9	18.4	5.3
	Ewing sarcoma	16	13	23.1	61.5	7.7	7.7
	Rhabdomyosarcoma	7	6	33.3	33.3	16.7	16.7
	Schwannoma	56	46	15.2	37.0	26.1	21.7
	Synovial sarcoma	11	10	10.0	60.0	20.0	10.0
	Osteosarcoma	17	13	69.2	30.8	0.0	0.0
	Chondrosarcoma	15	8	25.0	37.5	37.5	0.0
	Rhabdoid tumor	5	4	25.0	50.0	0.0	25.0

**Table 2:** **ANXA4 immunostaining and tumor phenotype.** Abbreviation: p = pathological, T = tumor stage, G = Grade, N = lymph node status, HER2 = Human Epidermal Growth Factor Receptor 2, ER = estrogen receptor, PR = progesterone receptor, ISUP = International Society of Urological Pathology, UICC = Union for International Cancer Control, M = distant metastasis, L = lymphatic invasion, V = venous invasion, MMR = mismatch repair, RAS = Rat Sarcoma, BRAF = B-rapidly accelerated fibrosarcoma.

			ANXA4 Immunostaining						
			n	n Missing	Negative (%)	Weak (%)	Moderate (%)	Strong (%)	*p*
**Invasive breast carcinoma of** **no special type**		All tumors	1159		54.8	6.3	13.0	25.9	
		pT1	525	142	49.5	5.7	12.8	32.0	**<0.0001**
		pT2	411		63.3	5.1	12.2	19.5	
		pT3-4	81		66.7	6.2	12.3	14.8	
		G1	92	111	51.1	4.3	12.0	32.6	**0.0018**
		G2	626		55.6	4.3	12.8	27.3	
		G3	330		58.2	9.7	11.8	20.3	
		pN0	220	755	39.1	6.4	17.3	37.3	**0.0247**
		pN+	184		53.8	6.5	16.3	23.4	
		HER2 negative	288	821	25.0	10.1	22.2	42.7	0.5100
		HER2 positive	50		28.0	18.0	16.0	38.0	
		ER negative	29	818	24.1	13.8	13.8	48.3	0.8359
		ER postive	312		26.3	11.5	21.8	40.4	
		PR negative	83	821	32.5	12.0	15.7	39.8	0.4211
		PR postive	255		23.1	11.8	23.1	42.0	
**Clear cell renal cell carcinoma**		All tumors	1365		0.1	0.4	1.0	98.6	
		ISUP 1	279	145	0.0	0.4	0.0	99.6	**0.0026**
		ISUP 2	557		0.0	0.4	0.7	98.9	
		ISUP 3	303		0.0	0.3	1.3	98.3	
		ISUP 4	81		1.2	1.2	3.7	93.8	
		Fuhrman 1	54	365	0.0	0.0	0.0	100.0	**0.0006**
		Fuhrman 2	611		0.0	0.2	0.2	99.7	
		Fuhrman 3	249		0.0	0.4	0.4	99.2	
		Fuhrman 4	86		0.0	1.2	5.8	93.0	
		Thoenes 1	316	546	0.0	0.0	0.0	100.0	**0.0135**
		Thoenes 2	414		0.0	0.5	0.7	98.8	
		Thoenes 3	89		0.0	1.1	3.4	95.5	
		UICC 1	292	903	0.0	0.3	0.3	99.3	**0.0140**
		UICC 2	33		0.0	3.0	3.0	93.9	
		UICC 3	74		0.0	0.0	0.0	100.0	
		UICC 4	63		0.0	1.6	4.8	93.7	
		pT1	823	16	0.0	0.4	0.6	99.0	0.2430
		pT2	139		0.0	0.7	0.7	98.6	
		pT3-4	387		0.3	0.3	1.6	97.9	
		pN0	319	1014	0.3	0.9	0.6	98.1	0.8850
		pN+	32		0.0	0.0	0.0	100.0	
		M0	95	1188	0.0	0.0	0.0	100.0	0.1804
		M1	82		0.0	1.2	3.7	95.1	
**Papillary renal cell carcinoma**		All tumors	373		0.0	0.5	1.6	97.9	
		ISUP 1	43	33	0.0	0.0	0.0	100.0	0.2886
		ISUP 2	203		0.0	1.0	1.5	97.5	
		ISUP 3	88		0.0	0.0	3.4	96.6	
		ISUP 4	6		0.0	0.0	0.0	100.0	
		Fuhrman 1	1	123	0.0	0.0	0.0	100.0	0.9750
		Fuhrman 2	166		0.0	0.0	0.6	99.4	
		Fuhrman 3	74		0.0	0.0	1.4	98.6	
		Fuhrman 4	9		0.0	0.0	0.0	100.0	
		Thoenes 1	53	163	0.0	0.0	0.0	100.0	0.9386
		Thoenes 2	141		0.0	0.0	0.7	99.3	
		Thoenes 3	16		0.0	0.0	0.0	100.0	
		UICC 1	92	250	0.0	0.0	0.0	100.0	1.0000
		UICC 2	15		0.0	0.0	0.0	100.0	
		UICC 3	5		0.0	0.0	0.0	100.0	
		UICC 4	11		0.0	0.0	0.0	100.0	
		pT1	264	7	0.0	0.8	1.9	97.3	0.5082
		pT2	55		0.0	0.0	0.0	100.0	
		pT3-4	47		0.0	0.0	2.1	97.9	
		pN0	79	275	0.0	1.3	2.5	96.2	0.8587
		pN+	19		0.0	0.0	0.0	100.0	
**Urothelial carcinoma of the urinary bladder**		All tumors	451		4.9	14.6	18.4	62.1	
		pTa G2 low	80	0	7.5	11.3	13.8	67.5	0.0644
		pTa G2 high	74		4.1	20.3	23.0	52.7	
		pTa G3	81		7.4	22.2	21.0	49.4	
		pTa	235	0	6.4	17.9	19.1	56.6	0.0767
		pT2-4	216		3.2	11.1	17.6	68.1	
		pT2	49	0	0.0	12.2	14.3	73.5	**0.0102**
		pT3	118		1.7	13.6	17.8	66.9	
		pT4	49		10.2	4.1	20.4	65.3	
	**pT2-4 only**	G2	15	0	6.7	20.0	20.0	53.3	0.7356
		G3	201		3.0	10.4	17.4	69.2	
		pN0	130	2	3.8	10.8	19.2	66.2	0.9145
		pN+	84		2.4	10.7	15.5	71.4	
		L0	126	0	4.0	11.9	18.3	65.9	0.9134
		L1	90		2.2	10.0	16.7	71.1	
		V0	174	0	1.7	9.8	18.4	70.1	0.1328
		V1	42		9.5	16.7	14.3	59.5	
**Pancreatic ductal adenocarcinoma**		All tumors	428		0.2	1.2	3.0	95.6	
		pT1	10	2	0.0	0.0	0.0	100.0	**0.0017**
		pT2	66		0.0	0.0	0.0	100.0	
		pT3	325		0.0	1.5	3.1	95.4	
		pT4	25		4.0	0.0	12.0	84.0	
		G1	15	21	0.0	0.0	0.0	100.0	0.2315
		G2	303		0.0	1.7	2.6	95.7	
		G3	89		0.0	0.0	5.6	94.4	
		pN0	93	2	0.0	0.9	1.9	97.2	0.7418
		pN+	333		0.0	1.1	3.4	95.5	
**Gastric adenocarcinoma**		All tumors	377		1.9	2.4	5.6	90.2	
		pT1-2	54	75	3.7	1.9	1.9	92.6	0.2475
		pT3	126		1.6	2.4	7.1	88.9	
		pT4	122		0.8	0.8	7.4	91.0	
		pN0	79	65	2.5	1.3	6.3	89.9	0.9673
		pN+	223		1.3	1.8	6.3	90.6	
		MMR proficient	253	83	0.4	1.6	4.7	93.3	0.0853
		MMR deficient	41		2.4	4.9	14.6	78.0	
**Hepatcellular carcinoma**		All tumors	133		1.5	5.3	9.8	83.5	
		pT1-2	58	6	1.7	6.9	5.2	86.2	0.1114
		pT3	43		2.3	4.7	11.6	81.4	
		pT4	26		0.0	0.0	19.2	80.8	
		pN0	33	95	0.0	6.1	18.2	75.8	0.6341
		pN+	5		0.0	0.0	0.0	100.0	
		G1	34	3	5.9	2.9	8.8	82.4	**0.0247**
		G2	63		0.0	1.6	12.7	85.7	
		G3	33		0.0	12.1	6.1	81.8	
**Adenocarcinoma of the colon**		All tumors	1845		1.3	2.3	3.4	93.0	
		pT1	62	44	4.8	1.6	1.6	91.9	**0.0098**
		pT2	384		0.8	2.6	2.1	94.5	
		pT3	1002		1.2	1.7	3.7	93.4	
		pT4	353		1.4	3.7	4.2	90.7	
		pN0	945	47	1.4	2.4	3.5	92.7	0.9840
		pN+	853		1.2	2.1	3.4	93.3	
		V0	1285	115	1.4	2.2	3.5	92.9	0.9168
		V1	445		1.1	2.9	3.4	92.6	
		L0	650	122	1.7	2.0	3.8	92.5	0.6581
		L1	1073		1.0	2.6	3.3	93.1	
		left side	804	730	2.0	3.0	4.0	91.0	0.9876
		right side	311		1.9	3.5	3.5	91.0	
		MMR proficient	1026	712	1.7	2.1	3.4	92.8	0.0586
		MMR deficient	107		1.9	2.8	10.3	85.0	
		RAS wildtype	462	1022	1.9	2.2	3.0	92.9	0.7698
		RAS mutation	361		1.9	1.1	3.9	93.1	
		BRAF wildtype	258	1512	1.9	0.8	4.3	93.0	0.5192
		BRAF V600E mutation	48		2.1	2.1	10.4	85.4	
**Endometrioid endometrial carcinoma**		All tumors	149		2.0	13.4	14.8	69.8	
		pT1	92	2	1.1	16.3	12.0	70.7	0.0872
		pT2	23		4.3	4.3	30.4	60.9	
		pT3-4	32		3.1	12.5	9.4	75.0	
		pN0	45	77	2.2	11.1	17.8	68.9	0.5956
		pN+	27		0.0	22.2	11.1	66.7	
**Serous high-grade carcinoma of the ovary**		All tumors	266		81.2	7.1	3.0	8.6	
		pT1	24	22	66.7	12.5	4.2	16.7	0.0753
		pT2	33		93.9	3.0	0.0	3.0	
		pT3	187		79.7	7.0	3.7	9.6	
		pN0	60	88	80.0	8.3	6.7	5.0	0.4227
		pN1	118		82.2	6.8	1.7	9.3	
**Papillary thyroid carcinoma**		All tumors	305		2.6	6.9	19.3	71.1	
		pT1	138	3	2.9	4.3	18.8	73.9	0.5018
		pT2	77		2.6	10.4	19.5	67.5	
		pT3-4	87		2.3	8.0	20.7	69.0	
		pN0	84	108	4.8	7.1	25.0	63.1	0.3756
		pN+	113		0.9	7.1	19.5	72.6	
**Testicular seminoma**		All tumors	281		89.3	8.5	1.1	1.1	
		pT1	178	5	89.9	7.9	1.1	1.1	0.0908
		pT2	64		85.9	14.1	0.0	0.0	
		pT3	34		91.2	2.9	2.9	2.9	
		V0	236	17	89.0	8.5	1.3	1.3	0.8300
		V1	28		89.3	10.7	0.0	0.0	
		L0	207	12	89.9	7.7	1.4	1.0	0.5327
		L1	62		85.5	12.9	0.0	1.6	
		Spermatic cord infiltration (no)	215	30	89.8	9.8	0.0	0.5	0.1088
		Spermatic cord infiltration (yes)	36		91.7	2.8	2.8	2.8	
		Rete testis infiltration (no)	121	15	90.1	8.3	0.8	0.8	0.9930
		Rete testis infiltration (yes)	145		89.0	9.0	1.4	0.7	

## Data Availability

All data generated or analyzed during this study are available from the corresponding author (R.S.) upon reasonable request.

## Supplementary Materials

The following supporting information can be downloaded at: https://www.mdpi.com/article/10.3390/cancers18193077/s1, Supplementary Table S1. ANXA4 immunostaining and tumor phenotype after correction for multiple comparison. Correction for multiple comparisons was performed using the Bonferroni method. Abbreviation: p = pathological, T = tumor stage, G = Grade, N = lymph node status, HER2 = Human Epidermal Growth Factor Receptor 2, ER = estrogen receptor, PR = progesterone receptor, ISUP = International Society of Urological Pathology, UICC = Union for International Cancer Control, M = distant metastasis, L = lymphatic invasion, V = venous invasion, MMR = mismatch repair, RAS = Rat Sarcoma, BRAF = B-rapidly accelerated fibrosarcoma. Supplementary Figure S1. Assay validation by comparison of antibodies. Using ARX-504, a strong ANXA4 immunostaining was seen in epithelial cells of the duodenum (A), intercalated and excretory ducts of the pancreas (B), and the luminal membrane of amnion cells of the placenta (C), while ANXA4 staining was predominantly seen in basal cells of the epidermis (D), weak in a subsets of cortical cells of the adrenal gland (E) and of the corpus luteum of the ovary (F), while ANXA4 staining was largely absent in the cerebrum (G) and the tubuli of the testis (H). Using the validation antibody 1A10B5, all staining patterns obtained by ARX-504 were confirmed in these tissues (a–g). The images (A–H,a–h) were obtained from consecutive tissue sections.

## Abbreviations

ANXA4	Annexin A4
HCC	hepatocellular carcinoma
IHC	immunohistochemistry
TMA	tissue microarray
pRCC	papillary renal cell carcinoma
NST	no special type
ccRCC	clear cell renal cell carcinoma
ANXA1	Annexin A1
