# Supplementary material for: Expression of Annexin A4 in Cancer: A Tissue Microarray Study Involving More than 17,600 Cancers from 148 Tumor Entities
Source: Cancers (Basel). 2026 Sep 22;18(19):3077. doi: 10.3390/cancers18193077 (PMC13663959; doi:10.3390/cancers18193077)
Supplement: Supplementary file 1 [file cancers-18-03077-s001.zip › cancers-4483010-supplementary.pdf]

Supplementary Figure 1. Assay validation by comparison of antibodies. Using ARX-504 a strong ANXA4 immunostaining was seen in epithelial cells of the duodenum (A), intercalated and excretory ducts of the pancreas (B) and the luminal membrane of amnion cells of the placenta (C) while ANXA4 staining was predominantly seen in basal cells of the epidermis (D), weak in a subsets of cortical cells of the adrenal gland (E) and of the corpus luteum of the ovary (F), while ANXA4 staining was largely absent in the cerebrum (G) and the tubuli of the testis (H). Using the validation antibody 1A10B5, all staining patterns obtained by ARX-504 were confirmed in these tissues (a-g). The images A-H and a-h were obtained from consecutive tissue sections.

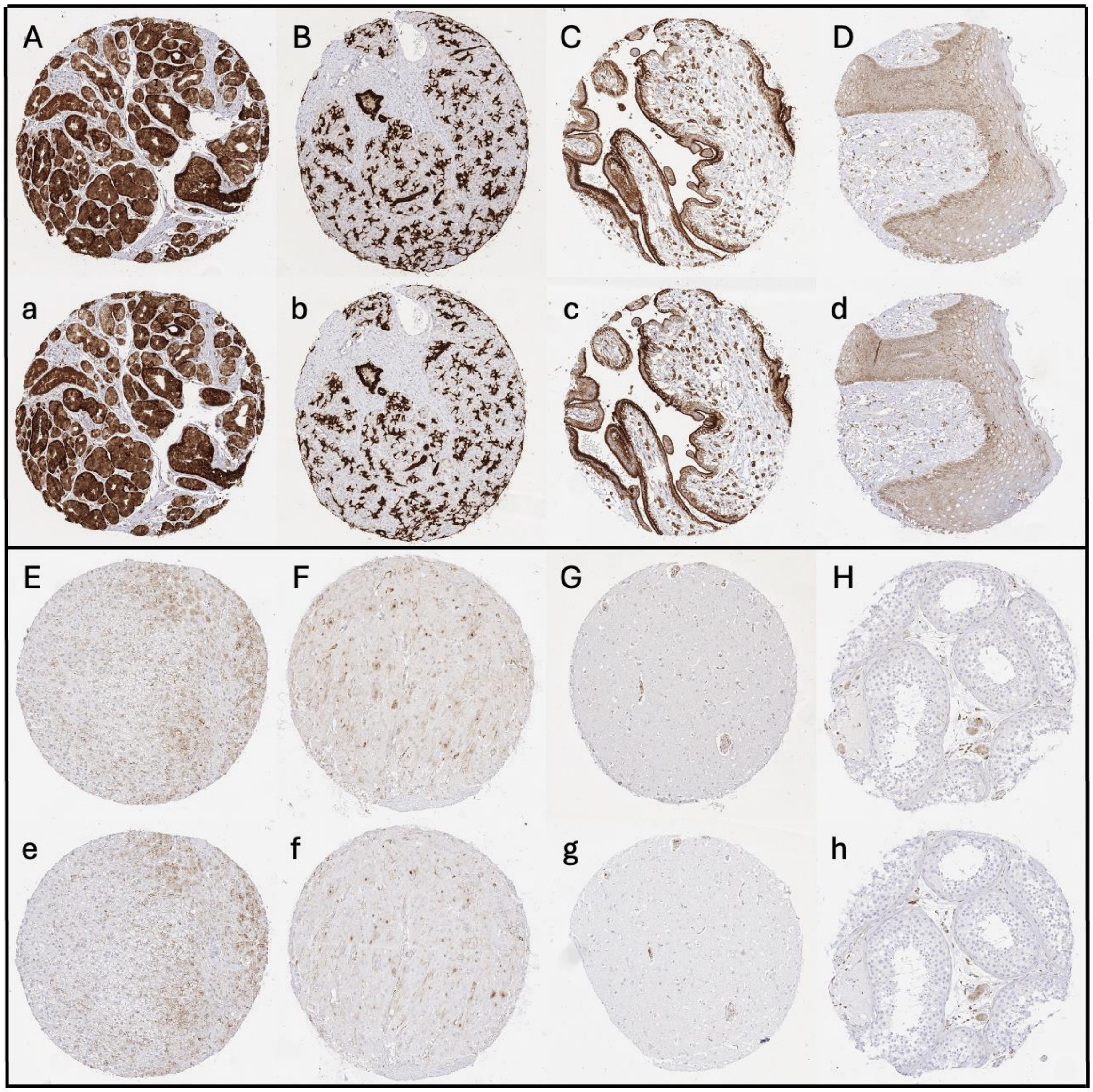

Supplementary Table 1. ANXA4 immunostaining and tumor phenotype after correction for multiple comparison. Correction for multiple comparisons was performed using the Bonferroni method. Abbreviation: p=pathological, T=tumor stage, G=Grade, N=lymph node status, HER2=Human Epidermal Growth Factor Receptor 2 , ER=estrogen receptor, PR=progesterone receptor, ISUP=International Society of Urological Pathology, UICC=Union for International Cancer Control, M=distant metastasis, L=lymphatic invasion, V=venous invasion, MMR=mismatch repair, RAS=Rat Sarcoma, BRAF=B-rapidly accelerated fibrosarcoma.

		ANXA4 immunostaining							
		n	n missing	negative (%)	weak (%)	moderate (%)	strong (%)	P	Bonferroni-adjusted P
Invasive breast carcinoma of no special type	All tumors	1159		54.8	6.3	13.0	25.9		
	pT1	525	142	49.5	5.7	12.8	32.0	**<0.0001**	**<0.0006**
	pT2	411		63.3	5.1	12.2	19.5		
	pT3-4	81		66.7	6.2	12.3	14.8		
	G1	92	111	51.1	4.3	12.0	32.6	**0.0018**	**0.0108**
	G2	626		55.6	4.3	12.8	27.3		
	G3	330		58.2	9.7	11.8	20.3		
	pN0	220	755	39.1	6.4	17.3	37.3	**0.0247**	0.1482
	pN+	184		53.8	6.5	16.3	23.4		
	HER2 negative	288	821	25.0	10.1	22.2	42.7	0.5100	1.0000
	HER2 positive	50		28.0	18.0	16.0	38.0		
	ER negative	29	818	24.1	13.8	13.8	48.3	0.8359	1.0000
	ER postive	312		26.3	11.5	21.8	40.4		
	PR negative	83	821	32.5	12.0	15.7	39.8	0.4211	1.0000
	PR postive	255		23.1	11.8	23.1	42.0		
Clear cell renal cell carcinoma	All tumors	1365		0.1	0.4	1.0	98.6		
	ISUP 1	279	145	0.0	0.4	0.0	99.6	**0.0026**	**0.0182**
	ISUP 2	557		0.0	0.4	0.7	98.9		
	ISUP 3	303		0.0	0.3	1.3	98.3		
	ISUP 4	81		1.2	1.2	3.7	93.8		
	Fuhrman 1	54	365	0.0	0.0	0.0	100.0	**0.0006**	**0.0042**
	Fuhrman 2	611		0.0	0.2	0.2	99.7		
	Fuhrman 3	249		0.0	0.4	0.4	99.2		
	Fuhrman 4	86		0.0	1.2	5.8	93.0		
	Thoenes 1	316	546	0.0	0.0	0.0	100.0	**0.0135**	0.0945
	Thoenes 2	414		0.0	0.5	0.7	98.8		
	Thoenes 3	89		0.0	1.1	3.4	95.5		
	UICC 1	292	903	0.0	0.3	0.3	99.3	**0.0140**	0.0980
	UICC 2	33		0.0	3.0	3.0	93.9		
	UICC 3	74		0.0	0.0	0.0	100.0		
	UICC 4	63		0.0	1.6	4.8	93.7		
	pT1	823	16	0.0	0.4	0.6	99.0	0.2430	1.0000
	pT2	139		0.0	0.7	0.7	98.6		
	pT3-4	387		0.3	0.3	1.6	97.9		
	pN0	319	1014	0.3	0.9	0.6	98.1	0.8850	1.0000
	pN+	32		0.0	0.0	0.0	100.0		
	M0	95	1188	0.0	0.0	0.0	100.0	0.1804	1.0000
	M1	82		0.0	1.2	3.7	95.1		

Papillary renal cell carcinoma		All tumors	373		0.0	0.5	1.6	97.9		
		ISUP 1	43	33	0.0	0.0	0.0	100.0	0.2886	1.0000
		ISUP 2	203		0.0	1.0	1.5	97.5		
		ISUP 3	88		0.0	0.0	3.4	96.6		
		ISUP 4	6		0.0	0.0	0.0	100.0		
		Fuhrman 1	1	123	0.0	0.0	0.0	100.0	0.9750	1.0000
		Fuhrman 2	166		0.0	0.0	0.6	99.4		
		Fuhrman 3	74		0.0	0.0	1.4	98.6		
		Fuhrman 4	9		0.0	0.0	0.0	100.0		
		Thoenes 1	53	163	0.0	0.0	0.0	100.0	0.9386	1.0000
		Thoenes 2	141		0.0	0.0	0.7	99.3		
		Thoenes 3	16		0.0	0.0	0.0	100.0		
		UICC 1	92	250	0.0	0.0	0.0	100.0	1.0000	1.0000
		UICC 2	15		0.0	0.0	0.0	100.0		
		UICC 3	5		0.0	0.0	0.0	100.0		
		UICC 4	11		0.0	0.0	0.0	100.0		
		pT1	264	7	0.0	0.8	1.9	97.3	0.5082	1.0000
		pT2	55		0.0	0.0	0.0	100.0		
		pT3-4	47		0.0	0.0	2.1	97.9		
		pN0	79	275	0.0	1.3	2.5	96.2	0.8587	1.0000
		pN+	19		0.0	0.0	0.0	100.0		
Urothelial carcinoma of the urinary bladder		All tumors	451		4.9	14.6	18.4	62.1		
		pTa G2 low	80	0	7.5	11.3	13.8	67.5	0.0644	0.4508
		pTa G2 high	74		4.1	20.3	23.0	52.7		
		pTa G3	81		7.4	22.2	21.0	49.4		
		pTa	235	0	6.4	17.9	19.1	56.6	0.0767	0.5369
		pT2-4	216		3.2	11.1	17.6	68.1		
		pT2	49	0	0.0	12.2	14.3	73.5	**0.0102**	0.0714
		pT3	118		1.7	13.6	17.8	66.9		
		pT4	49		10.2	4.1	20.4	65.3		
		G2	15	0	6.7	20.0	20.0	53.3	0.7356	1.0000
		G3	201		3.0	10.4	17.4	69.2		
	pT2-4 only	pN0	130	2	3.8	10.8	19.2	66.2	0.9145	1.0000
		pN+	84		2.4	10.7	15.5	71.4		
		L0	126	0	4.0	11.9	18.3	65.9	0.9134	1.0000
		L1	90		2.2	10.0	16.7	71.1		
		V0	174	0	1.7	9.8	18.4	70.1	0.1328	0.9296
		V1	42		9.5	16.7	14.3	59.5		
Pancreatic ductal adenocarcinoma		All tumors	428		0.2	1.2	3.0	95.6		
		pT1	10	2	0.0	0.0	0.0	100.0	**0.0017**	**0.0051**
		pT2	66		0.0	0.0	0.0	100.0		
		pT3	325		0.0	1.5	3.1	95.4		
		pT4	25		4.0	0.0	12.0	84.0		
		G1	15	21	0.0	0.0	0.0	100.0	0.2315	0.6945
		G2	303		0.0	1.7	2.6	95.7		
		G3	89		0.0	0.0	5.6	94.4		
		pN0	93	2	0.0	0.9	1.9	97.2	0.7418	1.0000
		pN+	333		0.0	1.1	3.4	95.5		
Gastric adenocarcinoma		All tumors	377		1.9	2.4	5.6	90.2		
		pT1-2	54	75	3.7	1.9	1.9	92.6	0.2475	0.7425
		pT3	126		1.6	2.4	7.1	88.9		
		pT4	122		0.8	0.8	7.4	91.0		
		pN0	79	65	2.5	1.3	6.3	89.9	0.9673	1.0000
		pN+	223		1.3	1.8	6.3	90.6		
		MMR proficient	253	83	0.4	1.6	4.7	93.3	0.0853	0.2559
		MMR deficient	41		2.4	4.9	14.6	78.0		

Hepatcellular carcinoma	All tumors	133		1.5	5.3	9.8	83.5		
	pT1-2	58	6	1.7	6.9	5.2	86.2	0.1114	0.3342
	pT3	43		2.3	4.7	11.6	81.4		
	pT4	26		0.0	0.0	19.2	80.8		
	pN0	33	95	0.0	6.1	18.2	75.8	0.6341	1.0000
	pN+	5		0.0	0.0	0.0	100.0		
	G1	34	3	5.9	2.9	8.8	82.4	**0.0247**	0.0741
	G2	63		0.0	1.6	12.7	85.7		
	G3	33		0.0	12.1	6.1	81.8		
Adenocarcinoma of the colon	All tumors	1845		1.3	2.3	3.4	93.0		
	pT1	62	44	4.8	1.6	1.6	91.9	**0.0098**	0.0784
	pT2	384		0.8	2.6	2.1	94.5		
	pT3	1002		1.2	1.7	3.7	93.4		
	pT4	353		1.4	3.7	4.2	90.7		
	pN0	945	47	1.4	2.4	3.5	92.7	0.9840	1.0000
	pN+	853		1.2	2.1	3.4	93.3		
	V0	1285	115	1.4	2.2	3.5	92.9	0.9168	1.0000
	V1	445		1.1	2.9	3.4	92.6		
	L0	650	122	1.7	2.0	3.8	92.5	0.6581	1.0000
	L1	1073		1.0	2.6	3.3	93.1		
	left side	804	730	2.0	3.0	4.0	91.0	0.9876	1.0000
	right side	311		1.9	3.5	3.5	91.0		
	MMR proficient	1026	712	1.7	2.1	3.4	92.8	0.0586	0.4688
	MMR deficient	107		1.9	2.8	10.3	85.0		
	RAS wildtype	462	1022	1.9	2.2	3.0	92.9	0.7698	1.0000
	RAS mutation	361		1.9	1.1	3.9	93.1		
	BRAF wildtype	258	1512	1.9	0.8	4.3	93.0	0.5192	1.0000
	BRAF V600E mutation	48		2.1	2.1	10.4	85.4		
Endometrioid endometrial carcinoma	All tumors	149		2.0	13.4	14.8	69.8		
	pT1	92	2	1.1	16.3	12.0	70.7	0.0872	0.1744
	pT2	23		4.3	4.3	30.4	60.9		
	pT3-4	32		3.1	12.5	9.4	75.0		
	pN0	45	77	2.2	11.1	17.8	68.9	0.5956	1.0000
	pN+	27		0.0	22.2	11.1	66.7		
Serous high-grade carcinoma of the ovary	All tumors	266		81.2	7.1	3.0	8.6		
	pT1	24	22	66.7	12.5	4.2	16.7	0.0753	0.1506
	pT2	33		93.9	3.0	0.0	3.0		
	pT3	187		79.7	7.0	3.7	9.6		
	pN0	60	88	80.0	8.3	6.7	5.0	0.4227	0.8454
	pN1	118		82.2	6.8	1.7	9.3		
Papillary thyroid carcinoma	All tumors	305		2.6	6.9	19.3	71.1		
	pT1	138	3	2.9	4.3	18.8	73.9	0.5018	1.0000
	pT2	77		2.6	10.4	19.5	67.5		
	pT3-4	87		2.3	8.0	20.7	69.0		
	pN0	84	108	4.8	7.1	25.0	63.1	0.3756	0.7512
	pN+	113		0.9	7.1	19.5	72.6		
Testicular seminoma	All tumors	281		89.3	8.5	1.1	1.1		
	pT1	178	5	89.9	7.9	1.1	1.1	0.0908	0.4540
	pT2	64		85.9	14.1	0.0	0.0		
	pT3	34		91.2	2.9	2.9	2.9		
	V0	236	17	89.0	8.5	1.3	1.3	0.8300	1.0000
	V1	28		89.3	10.7	0.0	0.0		
	L0	207	12	89.9	7.7	1.4	1.0	0.5327	1.0000
	L1	62		85.5	12.9	0.0	1.6		
	Spermatic cord infiltration (no)	215	30	89.8	9.8	0.0	0.5	0.1088	0.5440
	Spermatic cord infiltration (yes)	36		91.7	2.8	2.8	2.8		
	Rete testis infiltration (no)	121	15	90.1	8.3	0.8	0.8	0.9930	1.0000
	Rete testis infiltration (yes)	145		89.0	9.0	1.4	0.7		
